# Study protocol medical rehabilitation after COVID-19 disease: an observational study with a comparison group with obstructive airway disease / Re_Co

**DOI:** 10.1186/s12913-021-06378-4

**Published:** 2021-04-22

**Authors:** Mercedes Rutsch, Jördis Frommhold, Heike Buhr-Schinner, Levan Djeiranachvili, Thomas Gross, Per Otto Schüller, Alexander Katalinic, Ruth Deck

**Affiliations:** 1 Institute for Social Medicine and Epidemiology, Department Rehabilitation Sciences, University of Lübeck, Ratzeburger Allee 160, 23562 Lübeck, Germany; 2 MEDIAN Klinik Heiligendamm, Kinderstrand 1, 18209 Bad Doberan, Germany; 3 Ostseeklinik Schönberg-Holm, An den Salzwiesen 1, 24217 Schönberg-Holm, Germany; 4 Median Klinik Flachsheide Bad Salzuflen, Forsthausweg 1, 32105 Bad Salzuflen, Germany; 5 Reha-Zentrum Schömberg, Römerweg 50, 75328 Schömberg, Germany; 6 MEDIAN Klinik Flechtingen, Parkstraße, 39345 Flechtingen, Germany

**Keywords:** Coronavirus disease, COVID-19, Rehabilitation, Post‐acute

## Abstract

**Background:**

Novel coronavirus disease 2019 (COVID-19) has been the subject of a numerous research projects over the past year. In cases with a severe disease course or threatening long-term impairment due to disease, the German health care system offers insured persons the possibility of medical rehabilitation. In contrast to what was observed and expected at the beginning of the pandemic, COVID-19 patients with varying degrees of disease severity are represented in rehabilitation. To date, there is no common consensus on the content and aftercare of rehabilitation nor is there any knowledge about the short- and long-term effects of such a rehabilitation programme. In addition, these aspects were not considered with regard to the varying severity of the course of the disease. The present research project investigates this question.

**Methods:**

The study sample will consist of *N* = 350 rehabilitants after COVID-19 and a comparison group (CG) with *N* = 230 rehabilitants suffering from an obstructive respiratory disease. The participants will be recruited at five German rehabilitation facilities and undergo medical rehabilitation. This prospective, exploratory, multicentre, mixed-methods study will be evaluated as follows: (A) The quantitative portion includes questionnaires at different points in time (at the beginning and end of rehabilitation, after six and twelve months) and contains standardised measurement instruments. For example, participation limitations, quality of life, health status, fatigue, psychomental limitations and disorders, performance in different areas of life and ability to work are measured. (B) Qualitative interviews are held at different times (end of rehabilitation, after six and twelve months), and an expert workshop is conducted. Topics are rehabilitation content, satisfaction and aftercare as well as different outcomes on subjective health and participation impairments.

**Discussion:**

Studies on other indications have already shown that pneumological rehabilitation has positive effects. Thus, it is expected that an improvement in different dimensions will be observed at the end of rehabilitation in both groups. With regard to the different severities of COVID-19, this study evaluates the long-term developments. Subsequently, the authors will elaborate concrete recommendations for medical rehabilitation after different courses of disease with regard to existing pneumological rehabilitation concepts.

**Trial Registration:**

German Register of Clinical Trials, www.drks.de.Identifier: DRKS00023642; Registered: 01.12.2020.Date and version identifier: 08.04.2021; version 4.

## Background

COVID-19 is caused by the SARS-CoV-2 virus and is characterised by an unspecific, varying and diverse course of the disease, ranging from the lack of symptoms to severe pneumonia with lung failure and death [1]. The majority of patients have a mild to moderate course of disease (approximately 81 %) [2]. As a result, approximately 18 % were hospitalised in the first wave of the disease [3]. However, a moderate course of COVID-19 does not equate to a fast recovery. Patients with a severe course of the disease or those who have long-lasting symptoms require further treatment [4].

There are currently no uniform definitions of the long-term effects of COVID-19 (“long COVID”) [1]. Especially in very mild or asymptomatic courses, little is known to date. The Robert Koch Institute (RKI) refers to studies of SARS-CoV-1 infection to obtain information about the long-term consequences of COVID-19. According to this information, long-term physical and psychological consequences could be expected for COVID-19 patients. RKI refers to studies that report persistent impairment of lung function [5, 6], reduced physical performance [5, 6], reduced health-related quality of life [5, 7] and various mental and neuropsychiatric impairments, such as anxiety disorders, panic attacks, depression, post-traumatic stress disorder [7]. Such evidence for COVID-19 survivors is currently only found in a few studies with short- to medium-term follow-up. In an Italian study, 143 hospitalised COVID-19 patients were questioned 60 days after their first symptoms. Only 13 % of the respondents stated that they were completely symptom free. In contrast, 32 % reported one or two symptoms, and 55 % reported three or more symptoms. Half of the respondents (53 %) complained of fatigue, 43 % of dyspnoea, 27 % of joint pain and 22 % of chest pain [8]. In another study conducted by King’s College London, the UK Covid-19 Symptom Study App assessed the long-term effects of the disease [9]. One in ten users reported persistent symptoms even after three weeks. Symptoms included fatigue, headache, cough, loss of smell, sore throat, delirium and chest pain.

Due to suspected long-term consequences of COVID-19, the literature provides recommendations for physiotherapeutic treatment and early rehabilitation [10, 11] and calls for scientific studies on the topic of rehabilitation with COVID-19 [12]. Social reintegration after coronavirus disease should be of particular importance in the post-acute phase [13] as well as for patients with a mild disease course but long-lasting symptoms. Therefore, a multi-professional and interdisciplinary rehabilitation approach is recommended for moderate and severe disease progression [14, 15]. According to recent publications, there is a consensus that multimodal and interdisciplinary pneumological rehabilitation can be described as an effective therapeutic approach [14]. It is necessary to analyse the pulmonary, physical, psychosocial and cognitive dimensions of the disease within scientific studies [16] and to identify individual rehabilitation strategies based on this analysis [12].

Case reports in social networks and on public television show that the long-term effects of COVID-19 will have to be scientifically investigated in the future. These reports also demonstrate that persistent symptoms can also diverge from a linear course and vary in intensity and impairment over time. As a result, on the one hand, persons with a severe course of the disease can be found in the context of follow-up rehabilitation. On the other hand, patients with mild or moderate disease progression are impaired by long-lasting symptoms and can improve their occupational and social participation disorder through rehabilitation.

Given the novelty of the disease, there is no uniform approach to medical rehabilitation after COVID-19. Neither the rehabilitation content nor outcomes are known. In addition, the aftercare that is offered, recommended and received is also unknown. This observational study will provide recommendations for action, include a comparison group and consider the severity of COVID-19.

### Aims

The aim of our study is to describe the health status after COVID-19 in the context of medical rehabilitation and its medium- and long-term effects in different dimensions. For this purpose, the study examines the burden of disease and changes in health status under medical rehabilitation and after six and twelve months. Based on the experience of studies on pneumological rehabilitation, we expect positive effects on the general state of health and psychomental disorders at the end of rehabilitation. At follow-up, we expect that in addition to the outcomes already mentioned, rehabilitation success is represented in the dimensions of participation, quality of life, performance in various areas of life, ability to work, and subjective prognosis of employment.

Including rehabilitants from different clinics, it is possible to describe different therapeutic approaches, groups of patients and courses of disease. Statements are made about the extent to which existing rehabilitation approaches meet the needs of COVID-19 rehabilitants, the extent to which new rehabilitation therapy concepts have to be developed and how these are constituted. To date, it is unclear which requirements rehabilitants have with regard to aftercare, which recommendations rehabilitation clinics make and to what extent rehabilitants implement them.

The analyses also considered the severity of the disease course and compared the study results with a reference group with obstructive airway disease (COPD or bronchial asthma). Finally, the study will provide concrete recommendations for rehabilitation after COVID-19.

### Intervention

As an observational study, there is no intervention in the strict sense. The rehabilitants receive medical rehabilitation directly following hospitalisation or with greater time after SARS-CoV-2 infection. The duration of rehabilitation is 21 days with the possibility of extension. Given that the study participants are recruited from different clinics, the rehabilitation programmes differ in terms of therapy concept and structure.

At the MEDIAN Klinik Heiligendamm, the therapeutic elements of rehabilitation include general strengthening, mobilisation and normalisation of respiratory functions. This is intended to improve respiratory distress, respiratory muscle strength, pulmonary resilience, psychomental impairments and/or stress-induced mental disorders of the disease. The main components of the therapy include respiratory gymnastics with respiratory muscle training as well as dissolving phlegm. If required, group discussions (1–2 h per week) are offered to exchange experiences of coronavirus disease, relaxation training and supportive psychological counselling to cope with trauma, fear or depression.

The Reha-Zenrtum Schömberg treats patients in the pneumology department after severe respiratory insufficiency caused by a respiratory disease (including bronchial asthma and COPD) and after pneumonia. With this experience, the rehabilitation centre also receives COVID-19 patients for rehabilitation. The therapy contents include physical and balneotherapy, physiotherapy and exercise therapy, occupational therapy and psychological support.

Ostseeklinik Schönberg-Holm offers medical rehabilitation for various respiratory diseases. These illnesses range from bronchial asthma and COPD to the novel disease COVID-19. This facility also pursues a multimodal and holistic treatment approach during pneumological rehabilitation.

The comparison group is recruited in the MEDIAN Klinik Flechtingen and MEDIAN Klinik Flachsheide Bad Salzuflen as well as in the Ostseeklinik Schönberg-Holm and Reha-Zentrum Schömberg. The CG consists of COPD and asthma bronchial patients. These patients receive rehabilitation and aftercare according to the standards of the German Pension Insurance. The rehabilitants receive treatments comparable to those after COVID-19. Depending on their needs, the rehabilitation includes physical training (respiratory gymnastics, functional gymnastics, ergometer training, walking training, weight training, and treadmill training), respiratory physiotherapy, respiratory therapy, education, psychological interventions, nutritional therapy, counselling by social services and inhalation.

The particular therapeutic approaches are documented and described within the study.

## Methods

### Eligibility criteria

Rehabilitants after varying degrees of COVID-19 who are able to work will be recruited to the study. The rehabilitants may no longer remain in isolation. Participants are between 18 and 65 years old and provide written consent. Participants in the control group have a diagnosis of obstructive airway disease, such as bronchial asthma or COPD. This group will be similar based on age, sex and ability to work.

Individuals who do not provide consent and who do not have sufficient knowledge of German are excluded. Furthermore, rehabilitation may not be paid by German Social Accident Insurance.

### Study design

This is a prospective, explorative, longitudinal multicentre observational study without any specific intervention. Different methodological components (mixed-method study; see sections A and B) are included. The aim of the study is to obtain an overview of the problems associated with the new disease COVID-19 to derive recommendations for future clinical practice.

The Institute for Social Medicine and Epidemiology at the University of Lübeck leads the study. Consecutive recruitment will occur in five different rehabilitation clinics in Germany (MEDIAN Klinik Heiligendamm, Reha-Zentrum Schömberg, Ostseeklinik Schönberg-Holm, MEDIAN Klinik Flachsheide Bad Salzuflen, MEDIAN Klinik Flechtingen). Each clinic designates a study representative who recruits suitable rehabilitants during their first days in rehabilitation. If possible, the study representatives personally contacted the study participants and provides them with the information material and the declaration of consent.

### A: quantitative measures

To measure the long-term burden of COVID-19 and the effects of rehabilitation, the study includes four measurement points: at the beginning (t_0_), at the end of rehabilitation (t_1_), six (t_2_) and twelve months after rehabilitation (t_3_). A questionnaire with standardised and validated instruments will be completed by all study participants at each measurement point.

In the rehabilitation clinics, the participants of both groups are recruited consecutively at the same time until the planned number of cases is reached.

#### Outcomes

Based on the International Classification of Functioning, Disability and Health (ICF), the IMET [17] measures the *restrictions for social and work-related participation* of persons with a chronic disease [18, 19]. The IMET is a valid instrument and assesses the limitations of participation in nine relevant dimensions of daily life. On a scale from 0 to 10, the respondent ranks how high their limitations are in each dimension. A high score is associated with limited participation. Both, individual items and the cumulative score can be evaluated.

The VR-12 measures *health-related quality of life (HRQoL)* at the beginning of rehabilitation and at catamnesis. The VR-12 is based on the SF-36 and the Veterans Health Study. It covers eight health domains, and a total score is provided [20]. We use the translated and culturally adapted German version, which was validated in a German sample of inpatient rehabilitation patients [21].

Core symptoms of *depression, anxiety and stress* are assessed with the DASS. The instrument is mainly used in patients with pain, but it is also used for patients without pain-specific symptoms. The short version in Germany comprises 21 items and 7 items for the respective scales of depression, anxiety and stress. The individual scales of the DASS have sufficient to good reliability and have also been classified as valid [22].

The presence of a *generalised anxiety* disorder as well as its symptom severity is measured using the GAD-7 [23]. For this purpose, 7 items are used according to the most important diagnostic criteria of generalised anxiety disorder as defined in the Diagnostic and Statistical Manual of Mental Disorders (DSM-IV). The response options from “not at all” to “nearly every day” indicate how often the problem occurred in the last two weeks. The scale has good reliability and validity.

*Depressiveness* is measured with the PHQ-9 [24], which is the depression module of the Patient Health Questionnaire (PHQ-D) and contains nine questions. Each item captures one of the nine DSM-IV criteria of “major depression”. According to a large-scale meta-analysis, the PHQ-9 has very good specificity and sensitivity [25].

The COPD Assessment Test (CAT) [26] assesses the *effects of chronic obstructive pulmonary disease*. The CAT is a reliable instrument designed to measure the impact of COPD on health status and daily life [27]. A semantic six-point differential scale is used for the eight items. Cough, phlegm, tightness, breathlessness going up hills/stairs, activity limitation at home, confidence leaving home, sleep and energy are measured with one item each.

The Work Ability Score (WAS) of the Work Ability Index (WAI) measures the *work ability*. In numerous studies, this score provided acceptable values in terms of validity and reliability [28]. The higher the score on the scale from 0 to 10, the higher the ability to work.

The German version of the SPE scale is used to predict the *subjective prognosis of employment*. To divide the study participants into two risk groups, the respondents assess their occupational risk with three items from which an overall score is formed. The validity and reliability of the SPE scale was positively evaluated in a large sample [29].

Using individual items, *occupational changes* are measured, including job retention interventions and types of job loss [30].

*Fatigue* is measured with the EORTC-FA12, which has been validated in large international and multicentre studies [31]. With the help of twelve four-stage items (“not at all” to “very much”), physical, emotional and cognitive impairments caused by fatigue are assessed. In a German study validating the questionnaire with breast cancer patients [32], a high consistency of the overall scale was observed; the subscale also shows good to high internal consistency [32].

Single items without standardisation capture *COVID-19-associated life events*. Questions are asked about the death of a relative due to COVID-19, COVID-19-associated physical and/or social deprivation, loss of autonomy and loss of economic livelihood. Participants are also asked if they infected someone else, how they feel about it and if they feel that their general practitioner takes their symptoms seriously.

*Performance in different areas of life* is assessed with three scales from 0 to 10 for the areas of work, everyday life and leisure. The individual scales are valid instruments for determining performance limitations. A low sum score indicates a high level of performance limitations [30, 33].

To assess the extent of *physical activity*, questions are asked in conjunction with Menski’s Federal Health Surveys [34].

The moderating variables include comorbidity [35], risk factors and sociodemographic variables [36].

The rehabilitants receive the questionnaires at four measurement points. Some of the outcomes that are not likely to change during rehabilitation are only measured at the beginning of rehabilitation and at catamnesis. These are parameters that probably change only insignificantly during rehabilitation (e.g., comorbidities) or outcomes addressing everyday life and the domestic environment. Table 1 presents the set of core instruments used in the study.

**Table 1:** Core set of instruments used in the quantitative part of the study

Dimensions	Instruments	t_0_	t_1_	t_2_	t_3_
Restriction of Participation	IMET [17]	●		●	●
Quality of Life	VR12 [20]	●		●	●
Depression, Anxiety and Stress Scale	DASS [22]	●	●	●	●
Stress-Related Psychomental Limitations and Disorders	GAD-7 [23]; PHQ-9 [24]	●	●	●	●
Corona-associated Life Events	Single Items	●	●	●	●
Effects of Chronic Obstructive Pulmonary Disease	CAT [26]	●	●	●	●
Fatigue	EORTC-FA 12 [32]	●	●	●	●
General State of Health	Single Items	●	●	●	●
Performance in Various Areas of Life	QGmR [30]	●		●	●
Occupational Changes	Single Items from QGmR [30]			●	●
Occupational Situation	Single Items	●		●	●
Ability to Work	Work Ability Score, WAS [28]	●		●	●
Subjective Prognosis of Employment	SPE-Scale [29]	●		●	●
Moderating Variables					
Comorbidity	Single Items [35]	●		●	●
Risk Factors	Single Items	●	●	●	●
Sociodemographic Data	Single Items [36]	●		●	●

*t*_0_baseline/right before rehabilitation; *t*_1_ 3-week follow-up/right after rehabilitation; *t*_2_ 6 months after rehabilitation, *t*_3_ 12 months after rehabilitation

### B: qualitative measures

Telephone interviews and an expert workshop occur during the study. The guidelines for the telephone interview were pre-tested with COVID-19 rehabilitants. Rehabilitants provided feedback on the comprehensibility of the questions and the length of the interview.

Regarding telephone interviews, nine rehabilitants after COVID-19 were interviewed at three points in time: after medical rehabilitation, six and twelve months after rehabilitation. In the comparison group, eight interviews were held six months after rehabilitation. The interview participants varied in terms of sex, age and educational background.

 In the semi-structured interviews, the participants were asked about the course of their disease, the contents and goals of their rehabilitation, their satisfaction, their current disease burden, their social and occupational participation and their rehabilitation aftercare.

The expert workshop involves clinical staff, funding agencies and scientists. The topics of the workshop include the rehabilitation procedure, general conditions of rehabilitation after COVID-19, and existing pneumological therapy concepts in rehabilitation. Finally, recommendations for rehabilitation after COVID-19 are formulated.

Both the interviews and the expert workshop follow guidelines. The guidelines and the results of the qualitative research are discussed by the project team and at the brain trust “Arbeitsgruppe qualitative Methoden” (AQUAM). The interviews last approximately 30 min, while the expert workshop is correspondingly longer. With the agreement of all participants, the interviews and the workshop are recorded and transcribed word by word. The evaluation of data is based on qualitative content analysis.

### Data management

#### Patient documentation

Study participants are uniquely organised and documented in all clinics using an automated documentation template. All study representatives are briefed on the template before recruitment.

The included study participants receive an identification number (ID) to pseudonymise their personal data. The first number is used to classify whether the participant receives rehabilitation after COVID-19 or belongs to the comparison group. The second number of the ID refers to the rehabilitation clinic where the participant is recruited. The subsequent numbering corresponds to the number of recruited participants. Personal data are only documented in a separate Excel file; appropriate linkage occurs using the ID. The data will not be sent to the ISE until the personal contents are deleted. This collection includes only standardised documentation in the clinics. The data from the questionnaire are collected and kept separately from the ISE.

### Data entry and control

The data for the quantitative part of the study (A) are generated by standardised written questionnaires. The answers to the questionnaires are entered into SPSS 22.0 by a student assistant under the guidance of the research assistant. The entered data are assessed for random duplicate entries (5 − 10 %) by checking their validity and plausibility.

### Data monitoring

#### Data monitoring in the clinics is not planned

##### Case number calculation/sample size

To determine an appropriate sample size, it is assumed that the indicators used to characterise the sample should be described with sufficient precision. Sufficient precision should be applied to continuous indicators if the range of the 95 % confidence interval around a sample mean does not exceed a pre-specified value. For categorical variables, it is analogously assumed that the range of the 95 % confidence interval should not exceed a pre-specified value by a sample proportion. In the case of a continuous variable, the limits mentioned are set at +/- 5 points for a scale with a value range of 0 to 100. In the case of proportions, the range of the 95 % confidence interval should not exceed +/- 10 % points in the range of percentages from 10 to 40 %. The required width of the 95 % confidence interval is achieved with a continuous variable from a sample size of N = 100. In the case of a binary variable in the range between 10 and 40 %, the width of the 95 % confidence interval will be less than 20 % points for sample sizes of 100 or more. To compensate for smaller deviations in the required number of cases, a 20 % larger sample is taken compared to this net number of cases, i.e., N = 120 cases. We also expect a dropout rate of 20 % [37, 38]. Thus, the MEDIAN Klinik Heiligendamm will recruit N = 150, and the Reha-Zentrum Schömberg and the Ostseeklinik Schönberg-Holm will separately recruit N = 100 COVID-19 rehabilitants. For the CG, the MEDIAN Klinik Flechtingen and Bad Salzuflen will recruit N = 75 rehabilitation patients, separately, and the Reha-Zentrum Schömberg and Ostseeklinik Schönberg-Holm will recruit N = 40 rehabilitants per clinic.

### Statistical methods

#### Quantitative analysis

In the course of the study, no predefined hypotheses are confirmed. Rather, the rehabilitation groups are precisely characterised. Comparative analyses are used to determine the relevant differences between the subgroups and the comparison group. Accordingly, we mainly provide descriptive evaluations on the burden of disease as well as on health-related changes during and after rehabilitation.

For the interval-scaled variables, variance analyses with measurement repetition are calculated to evaluate the pre-post differences of the outcomes at the various measurement times. Depending on the data constellation, the t-test for dependent or independent samples is used as a parametric significance test. Non-parametric tests are performed for ordinally scaled data. Subgroup analyses will be planned for sex, age and education as well as risk factors and comorbidities. To classify the findings in COVID-19 patients, the stress manifestations and the measured changes are compared with those in the CG.

In addition to the analysis of the health-related changes in relation to a comparison group, the treatment concepts from different rehabilitation facilities are also examined. The type and extent of the therapy, characteristics of the included rehabilitants, perceived benefits of the rehabilitation and subjectively experienced health improvements are recorded. All calculations are performed with SPSS 22.0.

### Qualitative analysis of the interviews and expert workshop

The interviews and focus group are analysed using qualitative content analysis [39–41]. We use MAXQDA 12 for coding. The transcripts are checked for relevant topics and interesting content to finally develop a category system. The main topics are derived from the research questions and the interview guide; subcategories are induced from materials developed, e.g., in subsumption [40]. To test the category system, independent test coding is performed by two researchers; then, the categories and definitions are modified. The entire coding process is developed in the form of a consensual coding [42], i.e., the transcriptions are coded independently by two scientists. Finally, the coding is consolidated to develop a consensus.

## Discussion

The novelty of the disease and the increasing use of rehabilitation services by COVID-19 patients enhances the call for research. At present, COVID-19 rehabilitation does not follow uniform guidelines. Therefore, it is necessary to review the different therapeutic approaches and to formulate recommendations for pneumological rehabilitation after COVID-19. Furthermore, the rehabilitation needs of COVID-19 patients and their rehabilitation capacity will be assessed. The concrete short-, medium- and long-term effects of COVID-19 medical rehabilitation have not yet been evaluated.

When assessing rehabilitation, it is also essential to analyse rehabilitation aftercare. To date, there is no evidence on the needs at the end of rehabilitation, nor is there a uniform approach to rehabilitation aftercare. This study clarifies which aftercare recommendations are given to these rehabilitants, to what extent they are realised and how the needs of the rehabilitants are met.

It now appears that COVID-19 survivors of any disease severity seek rehabilitation. Therefore, the study results are also differentiated against the different degrees of severity and additionally contrasted with a comparison group. Finally, recommendations for rehabilitation and rehabilitation aftercare need to be developed for COVID-19 patients.

### Innovation factor

 During the telephone interviews and written survey, the study participants are asked about their aftercare recommendations, the use of aftercare programmes and their wishes or needs for the time after rehabilitation. By classifying the results on the basis of a comparison group, conclusions are drawn for further projects on suitable aftercare strategies in the novel indication COVID-19. Further study could aim at maintaining long-term rehabilitation effects and maintaining or restoring employability. In addition, it may be possible that different recommendations are made for the varying severity of the disease course; therefore, further consideration is necessary.

### Immediate expected results of the project

As this is an observational study, the baseline situation and the changes in the mentioned outcomes are described. These results are reported in relation to the comparison group, and statements on the extent to which there are similarities and differences are provided. The rehabilitation contents, needs and ability; aftercare recommendations and their implementation are also described descriptively for different degrees of disease severity.

Although the rehabilitants do not receive an intervention in the strict sense, they all undergo rehabilitation. Based on previous studies on pneumological rehabilitation as well as from the current experience of rehabilitation physicians, we expect positive changes in outcomes during rehabilitation. For example, we expect a significant reduction in the burden of disease and psychological impairments at the end of rehabilitation. The extent to which the effects on outcomes change over all four measurement points will be described in this study.

The results of the study will show whether rehabilitants have different needs in terms of rehabilitation, its contents and aftercare due to their individual course of illness, their previous illnesses or other personal prerequisites. With regard to aftercare, it is assumed that individual needs range from their own initiative and classic aftercare programmes to more intensive aftercare.

### Transferability of the project results to the everyday care situation

In recent months, it has become apparent that the need for rehabilitation services for COVID-19 survivors is increasing. To offer a treatment that meets the needs of rehabilitants, appropriate research is necessary. At the end of the present study, practical recommendations for rehabilitation after COVID-19 will be developed. A common consensus in the rehabilitation procedure after COVID-19 can guarantee a universal standard in German rehabilitation.

The observational study is conducted in five German rehabilitation clinics. However, it is possible to transfer the results and provide recommendations to other pneumological rehabilitation clinics.

### Limitations

To ensure equal treatment, all characteristics of the participants (e.g., age, education) and the disease (e.g., severity of the disease, disease status, and comorbidity) are documented and compared between groups. If differences arise between the two groups, they are comparably integrated in the analysis (e.g., as covariates in multivariate methods or by propensity score matching). Based on our present knowledge, we assume that structural changes in the context of this project are very unlikely.

## Data Availability

The datasets generated and/or analysed during the current study are not publicly available due to the data protection regulations and declarations of consent of the study participants. The declarations of consent exclude the release of the data for external analyses. A sample declaration of consent is available from the corresponding author on request. The study results are published and thus made available to all those interested in the study. The study protocol, the dataset at the participant level and the statistical code for generating the results are not publicly available.

## Abbreviations

CG: Comparison group
COPD: Chronic obstructive pulmonary disease
COVID-19: Coronavirus disease
DRV Bund: Deutsche Rentenversicherung Bund
DSM-IV: Diagnostic and Statistical Manual of Mental Disorders
ICF: International Classification of Functioning, Disability and Health
ID: Identification number
IPD: Individual participant data
PHQ-D: Patient Health Questionnaire
PSM: Propensity score matching
